# Mapping ubiquitination sites of *S. cerevisiae* Mcm10

**DOI:** 10.1016/j.bbrep.2016.09.003

**Published:** 2016-09-19

**Authors:** Tianji Zhang, Brandy L. Fultz, Sapna Das-Bradoo, Anja-Katrin Bielinsky

**Affiliations:** a Department of Biochemistry, Molecular Biology and Biophysics, University of Minnesota, Minneapolis, MN 55455, United States; b Department of Natural Sciences, College of Science and Health Professions, Northeastern State University, 3100 East New Orleans Street, Broken Arrow, OK 74014, United States

**Keywords:** DNA replication, Mcm10, PCNA, Ubiquitination, 9-1-1 checkpoint clamp

## Abstract

Minichromosome maintenance protein (Mcm) 10 is a part of the eukaryotic replication machinery and highly conserved throughout evolution. As a multivalent DNA scaffold, Mcm10 coordinates the action of proteins that are indispensable for lagging strand synthesis, such as the replication clamp, proliferating cell nuclear antigen (PCNA). The binding between Mcm10 and PCNA serves an essential function during DNA elongation and is mediated by the ubiquitination of Mcm10. Here we map lysine 372 as the primary attachment site for ubiquitin on *S. cerevisiae* Mcm10. Moreover, we identify five additional lysines that can be ubiquitinated. Mutation of lysine 372 to arginine ablates ubiquitination of overexpressed protein and causes sensitivity to the replication inhibitor hydroxyurea in cells that are S-phase checkpoint compromised. Together, these findings reveal the high selectivity of the ubiquitination machinery that targets Mcm10 and that ubiquitination has a role in suppressing replication stress.

## Highlights

• *S. cerevisiae* Mcm10 is ubiquitinated at lysine 372.
• Alternative ubiquitination sites exist at five specific lysine residues.
• The *mcm10-K372R* mutation synergizes with a 9-1-1 checkpoint deficiency.

## 1 Introduction

Minichromosome maintenance protein (Mcm) 10 is an essential replication factor required for origin unwinding and DNA synthesis initiation. Multiple independent studies have identified Mcm10 as a major protector of genome integrity [1], [2], especially at common fragile sites [3]. Mcm10 binds to replication origins at the end of G1 phase after the Mcm2-7 core helicase has been loaded onto chromatin as an inactive double-hexamer [4], [5], [6]. Mcm10 directly interacts with Mcm2-7 dimers and facilitates the remodeling into monomers [7], [8]. This process is likely driven by Mcm10's DNA binding activity [9]. The change in Mcm2-7 conformation and the assembly of the mature replicative helicase, the Cdc45:Mcm2-7:GINS (CMG) complex, occur in early S phase through the coordinated action of cell cycle regulated kinases [10], [11]. For the duration of DNA replication Mcm10 stays attached to Mcm2-7 and serves as a DNA binding scaffold that links the CMG complex to the polymerization machinery [4]. Other interaction partners at replication forks include the chromosome transmission fidelity protein 4 (Ctf4), DNA polymerase-α (pol-α), the replication clamp, proliferating cell nuclear antigen (PCNA), and the 9-1-1 checkpoint clamp [2]. Mcm10 is required to maintain some of these key factors on chromatin [12]. It is therefore not surprising that partial loss of Mcm10 triggers replication stress in both *S.cerevisiae* and human tissue culture cells. Under those conditions, replication forks move slowly and elicit responses triggered by the accumulation of single-stranded (ss) DNA, a byproduct of fork stalling [13]. In budding yeast, this is counteracted by the activity of a small ubiquitin-like modifier (SUMO) targeted ubiquitin ligase, the action of which facilitates mitotic progression in the presence of incompletely replicated chromosomes [14].

Ubiquitination also regulates the binding between Mcm10 and PCNA, which is dependent on the cell cycle and a PCNA interacting peptide (PIP) box that is buried in the central domain of Mcm10 [15]. Curiously, the PIP box is part of a highly conserved oligonucleotide/-saccharide binding (OB) fold. These β-barrel motifs are common in RNA and DNA binding proteins and form a cleft that allows for the direct interaction with the nucleic acid backbone [16]. Perpendicular to the PIP box, which resides on the third β-sheet of the OB-fold, is a hydrophobic patch, the Hsp10-like domain that has been implicated in the binding of pol-α [9]. Due to the spatial arrangement of the PIP box and the Hsp10-like motifs, the simultaneous interaction of Mcm10 with pol-α and PCNA seems highly unlikely. Indeed, co-immunoprecipitation experiments revealed that Mcm10 has to be ubiquitinated in order to interact with PCNA, whereas pol-α physically associates only with the unmodified form of Mcm10 [15]. A previous study had suggested that two lysines of Mcm10 are mono-ubiquitinated [15]. Here, we identify lysine (K) 372 as the primary site and map several alternative sites for ubiquitin attachment on Mcm10 in *S. cerevisiae*.

## 2 Materials and methods

### 2.1 Strains and plasmids

Yeast strains used in this study are isogenic derivatives of W303. To overexpress wild type Mcm10 or Mcm10 mutants, cells were transformed with galactose inducible plasmids expressing histidine (His) and hemagglutinin (HA) tagged Mcm10. A copper inducible ubiquitin plasmid (YEp105) was transformed to overexpress ubiquitin [15]. To express the *MCM10* transgene under the control of its endogenous promoter, the coding sequence and 372 bp of upstream promoter sequence were cloned into pRS316 [17]. To express the C-terminally tagged 3HA- and His_8_-Mcm10 from the endogenous locus, pRS406 integration plasmids were used, which contained the 3′ half of the *MCM10* gene with the respective epitope tags. pRS406 constructs were linearized and transformed into the desired strains. Point mutations were introduced by a QuikChange Lightning Site Directed Mutagenesis Kit (Agilent Technologies). All strains used in this study were verified by DNA sequencing of genomic DNA and are listed in Table S1.

### 2.2 Protein overexpression and nickel affinity purification

Ubiquitin expression was induced with 100 μM Copper from the beginning of log phase (OD=0.2) to mid-log phase (OD=0.6) [18]. 2% galactose was added to induce Mcm10 expression at mid-log phase and cells were grown for 3 h. Cells were harvested and lysed with 1.85 M NaOH and 7.5% β-mercaptoethanol. Total protein was precipitated with 55% percent trichloroacetic acid (TCA) and resuspended in buffer A (8 M urea, 300 mM NaCl, 0.5% NP-40, 50 mM Na_2_HPO_4_, and 50 mM Tris). Lysate was bound to Ni-NTA beads (QIAGEN) at a ratio of 100 μl slurry per 1 mg protein. After overnight binding, three separate washes were applied with buffer A. The first wash was carried out with 10 bead volumes of buffer A’ (pH=8). The second wash was applied with 10 bead volumes of buffer A’ (pH=6.3). The third wash was performed with 10 bead volumes of buffer A’ (pH=6.3) to which 10 mM imidazole was added. Protein was eluted with 5 bead volumes of buffer B (8 M urea, 200 mM NaCl, 2% SDS, 50 mM Na_2_HPO_4_, 10 mM EDTA, 50 mM Tris pH=4.3). Eluates were concentrated to 500 µl-1 mL with Amicon filter units and fractionated on SDS-PAGE.

### 2.3 In-gel trypsin digestion

Comassie Brilliant Blue stained bands of interest were cut into 1×1 mm pieces and destained with destaining solution (25 mM ammonium bicarbonate, 50% acetonitrile) for 1 h. Samples were then treated with reducing buffer (25 mM ammonium bicarbonate, 50 mM (Tris [2-carboxyethyl] phosphine) for 1 h, and alkylation buffer (25 mM ammonium bicarbonate, 100 mM iodoacetamide) for 1 h. Alkylated gel pieces were washed with 25 mM ammonium bicarbonate and digested with 10 μg/μl trypsin overnight at 37 °C. Bands of interest were extracted by 1.67% formic acid and 66.7% acetonitrile, lyophilized and sent to the Center for Mass Spectrometry and Proteomics at the University of Minnesota for mass spectrometry analysis.

### 2.4 Mass spectrometry analysis

The peptide mixtures were desalted using the Stage Tip protocol [19] and approximately 1 μg was injected into a capillary liquid chromatography (LC) column online with a mass spectrometer. We analyzed the samples on an Orbitrap XL system with LC-tandem mass spectrometry (LC-MS/MS) with the following MS acquisition specifications: data dependent acquisition (DDA) was performed on the top 6 most intense ions from MS1 scans, ion trap automatic gain control setting was 3000 and the dynamic exclusion time period was 20 s. We analyzed two samples from a replicate experiment on an Orbitrap Velos system as described previously [20] with the following exceptions to the MS acquisition method: DDA on the top 10 most intense peaks detected in MS1 mode with CID (collision induced dissociation) activation at 35% normalized collision energy and 30 msec activation time, automatic gain control at 1 × 10E4 and 100 msec maximum injection time; dynamic exclusion duration was 30 s.

### 2.5 Data base searching

We used two data base search programs for tandem mass spectral interpretation. MSconvert (http://proteowizard.sourceforge.net/) was used for the conversion of .RAW files to mzXML files. mzXML files were converted to DTA files with an in-house tool (https://github.com/jmchilton/tint/). We searched the spectra with Sequest v27 rev12 (Thermo Fisher, San Jose, CA) [21] against the NCBI RefSeq yeast protein database from 2011 concatenated with a protein contaminants database in a combined forward and reversed fashion for false discovery rate calculations (13388 total entries). Search parameters were: precursor mass tolerance 0.1 Da, product ion mass tolerance 0.8 Da, fixed modification carbamidomethyl cysteine, variable modifications methionine oxidation (15.9949), lysine ubiquitination (114.0429) and 2 missed cleaves per peptide. Criteria for protein identification were as follows:

Scaffold (version Scaffold_4.3.4, Proteome Software Inc., Portland, OR) was used to validate MS/MS based peptide and protein identifications. Peptide identifications were accepted if they could be established at greater than 95% probability by the Peptide Prophet algorithm [22]. Protein identifications were accepted if they could be established at greater than 95% probability and contained at least 1 identified peptide. Protein probabilities were assigned by the Protein Prophet algorithm [23]. Proteins that contained similar peptides and could not be differentiated based on MS/MS analysis alone were grouped to satisfy the principles of parsimony. We also used PEAKS® Studio software [24] v 7.0 (Bioinformatics Solutions, Inc, Waterloo, ON CA) for interpretation of tandem mass spectra and protein inference. We used the following parameters in PEAKS®: merge spectra within 15 ppm precursor mass tolerance and 0.2 min retention time, correct precursors between charge states 2 and 9, filter quality setting >0.5; de novo precursor mass error 20 ppm and fragment mass error 0.8 Da, trypsin specificity, fixed modification carbamidomethyl cysteine, variable modification oxidation of methionine, 3 maximum variable modifications; database search precursor mass error 50 ppm and fragment mass error 0.8 Da, enzyme and standard modification settings identical to de novo parameters, maximum 3 missed cleave sites, maximum 4 variable modifications per peptide, NCBI RefSeq yeast database (6/18/13) combined with contaminant proteins (http://www.thegpm.org/cRAP/index.html) and FDR estimation enabled; PTM module parameters included: pyro-glutamic acid (−17.0265), N and Q deamidation (0.9840), M dioxidation (31.9898), K ubiquitination as GG (114.0429). Only peptides were considered that were identified more than once, unless noted otherwise.

### 2.6 Protein preparation and immunoblotting

Protein extract was prepared from asynchronous yeast cultures. Total protein was obtained by trichloroacetic acid (TCA) precipitation, fractionated by SDS-PAGE and subsequently transferred onto nitrocellulose membrane. HA- or His-tagged Mcm10 was visualized using a horseradish peroxidase (HRP)-conjugated anti-HA (Roche 3F10). LexA tagged Mcm10 was detected with an anti-LexA antibody (Abcam, ab14553).

### 2.7 β-galactosidase activity

Yeast two-hybrid assays were performed as described [15]. Briefly, 10 mL overnight cultures of cells harboring the indicated bait and prey plasmids were collected, washed first with 25 mL of ice-cold buffer P (50 mM sodium phosphate, pH 7.7, 300 mM sodium acetate, 10% glycerol, 1 mM 2-mercaptoethanol, 500 nM dithiolthreitol, 1 µg/mL pepstatin, 1 mM PMSF, 1 mM benzamidine, 0.5 µg/mL leupeptin), then a second time with 0.5 mL buffer P. Cells were resuspended in ~200 µL buffer P and subsequently lysed by vortexing with acid-washed glass beads. Extracts were clarified by centrifugation and the supernatant was tested for β-galactosidase activity using a β-galactosidase activity assay kit (Invitrogen). β-galactosidase activity and total protein, measured by a Bradford assay, were determined in triplicate from at least three individual clones for each two-hybrid strain. The interaction between Pol32 and PCNA served as a positive control.

### 2.8 Serial dilution assay

Yeast strains were grown for 2 days to saturation. 2 × 10^7^ cells were resuspended in 300 µL sterile water and pipetted into the first column of a 96-well plate. Serial, ten-fold dilutions were made in each successive column of the plate. Cells were transferred from the plate to the indicated growth medium using a 48-spoke inoculating manifold, and the plates were grown for 2 days at different temperatures as indicated.

## 3 Results

### 3.1 Mcm10 is mono-ubiquitinated at two lysines, K85 and K372

**Fig. 1:**
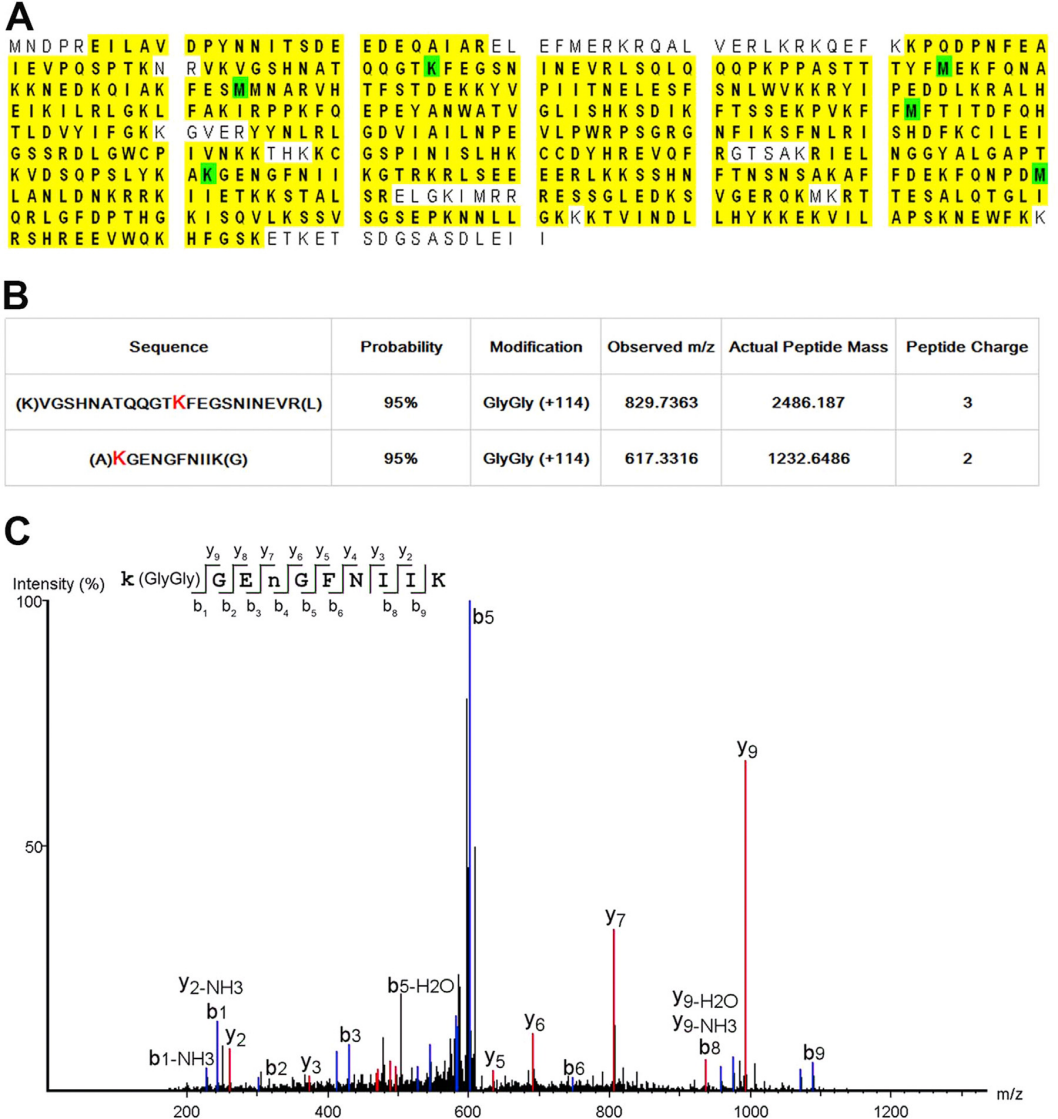
Mcm10 is ubiquitinated at lysines 85 and 372. Spectra identified by LC-MS/MS were analyzed by SEQUEST. (A) Peptide coverage of Mcm10 was 88%. Yellow colored regions show peptide sequences that were identified by MS. Green colored amino acids indicate modified residues; methionines (M) were oxidized, lysines (K) were ubiquitinated. (B) Peptides containing ubiquitinated lysines 85 (top) and 372 (bottom) were identified by a 114 Da mass shift. (C) B- and y- type ions after fragmentation of the lysine 372 containing peptide are shown. Ions observed indicate that lysine 372 is modified with the di-glycine remnant.

Our laboratory previously reported that Mcm10 exists in an unmodified and ubiquitinated form. The interaction with PCNA depends on di-ubiquitination of Mcm10, which we showed has mono-ubiquitin attached to two distinct lysines [15]. To determine which lysines of Mcm10 are ubiquitinated, His-tagged Mcm10 and ubiquitin were co-overexpressed and the tagged protein was isolated by Ni-NTA affinity purification under denaturing conditions. The eluate was fractionated by SDS-PAGE and the band containing modified Mcm10 was cut from the gel, digested with trypsin and analyzed by MS (Fig. 1). Since trypsin cleaves peptide chains mainly at the carboxyl side of the amino acids lysine or arginine (except when followed by proline), cleavage at the C terminus of conjugated ubiquitin leaves the signature of a 114 Da mass shift (Gly-Gly). The two lysines that carry this signature are K85 and K372. The result was confirmed in two separate pull-down experiments.

### 3.2 K-to-R substitutions at residues 85 and 372 of Mcm10 do not abolish the interaction with PCNA

**Fig. 2:**
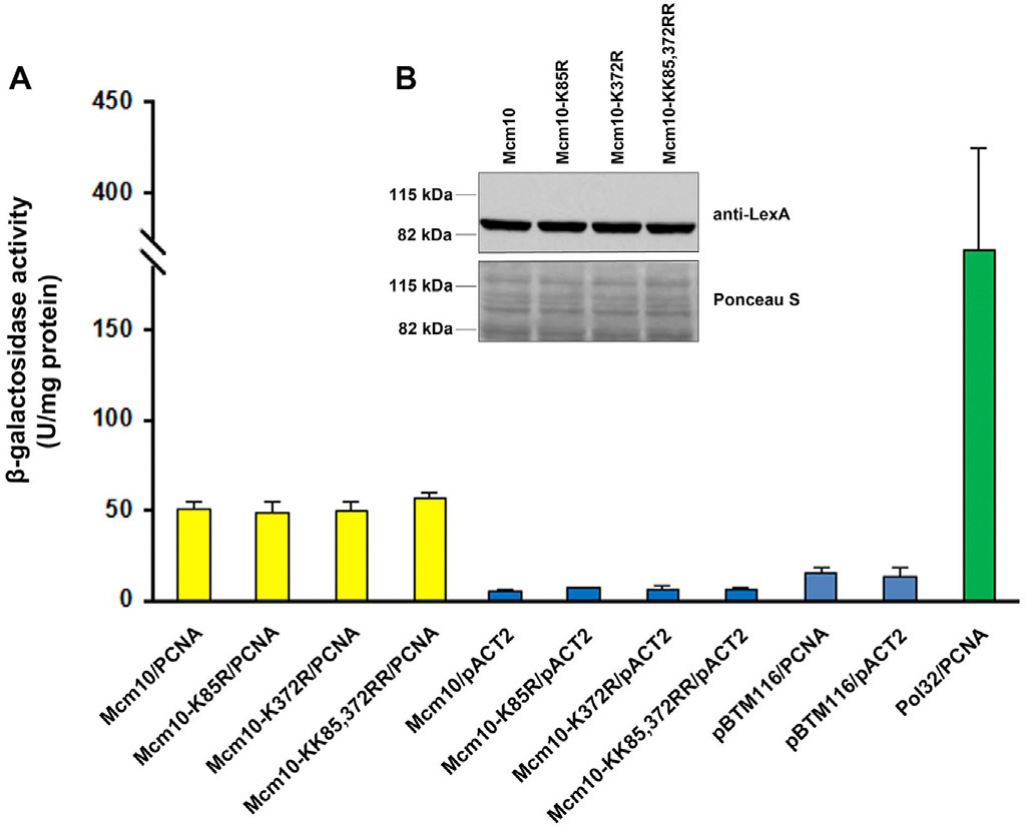
Mcm10 lysine mutants interact with PCNA in yeast two-hybrid assays. (A) β-galactosidase activity was measured in cell extracts obtained from yeast two-hybrid strains expressing Mcm10-, Mcm10-K85R-, Mcm10-K372R- and Mcm10-KK85,372RR-LexA fusion proteins that co-expressed PCNA. Pol32 and PCNA served as a positive control, and extracts expressing the pGAD2F and pBTM116 empty vectors served as negative controls. Each combination was tested in triplicate with three individual transformants. Error bars indicate standard deviations. (B) Immunoblot using a LexA-specific antibody to detect expression of wild-type Mcm10 and three lysine mutants. Ponceau S staining served as a loading control.

To test whether K-to-R substitutions at residues 85 or 372 of Mcm10 had any effect on PCNA binding, we generated single and double mutants as LexA fusion proteins and examined their interaction with PCNA by yeast two-hybrid assays. Binding was quantified by β-galactosidase activity. Wild-type Mcm10 showed a similar binding activity to the one reported previously. All mutant constructs were expressed at wild-type levels, however, we were unable to detect changes for any of the three mutants we generated (Fig. 2). Please note that the LexA antibody does not pick up the ubiquitinated form of Mcm10.

### 3.3 K-to-R mutants of Mcm10 are ubiquitinated at alternative sites

**Fig. 3:**
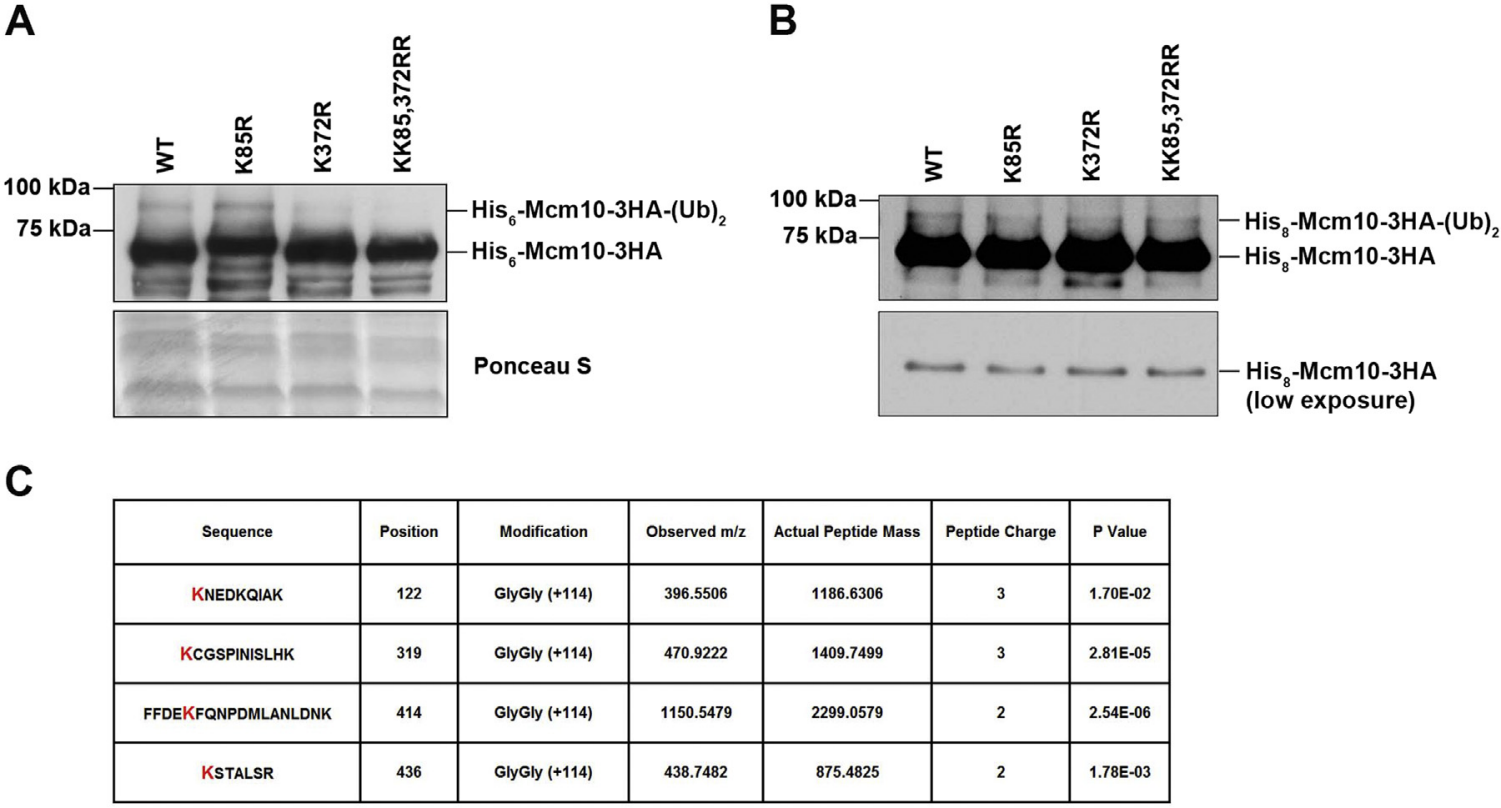
Lysine 372 is the primary ubiquitination site of Mcm10, but alternative lysines can serve as ubiquitin attachment sites. (A) Strains co-overexpressing wild-type or mutant forms of Mcm10 and ubiquitin were grown in medium lacking uracil and trytophan with raffinose as the carbon source. His-tagged Mcm10-3HA was induced by galactose and ubiquitin by copper. His-tagged Mcm10 was isolated by Ni-NTA affinity purification and fractionated by SDS-PAGE. Mcm10 (unmodified and di-ubiquitinated) was analyzed by western blot with an anti-HA antibody. Ponceau S staining served as a loading control. (B) Strains that expressed His-tagged Mcm10-3HA from the endogenous *MCM10* locus were grown in medium lacking trytophan with glucose as the carbon source. Ubiquitin overexpression was controlled by copper induction. His-tagged Mcm10 was isolated by Ni-NTA affinity purification and fractionated with SDS-PAGE. Mcm10 (unmodified and di-ubiquitinated) was analyzed by western blot with an anti-HA antibody. The top panel was overexposed to visualize the di-ubiquitinated Mcm10. The lower exposure (bottom) served as a loading control. (C) Summary of additional lysines besides K85 and K372 that carried a 114 Da Gly-Gly mass shift identified by PEAKS. The same LC-MS/MS data as in Fig. 1 was analyzed. Specific lysines that were ubiquitinated are shown in red. The mass shift was identified in at least one out of two experiments.

Because PCNA interacts exclusively with ubiquitinated Mcm10, we predicted that the K-to-R Mcm10 mutants were ubiquitinated at other residues besides 85 and 372. This has been commonly observed for ubiquitination events that lead to subsequent degradation, whereas the attachment of ubiquitin that serves to facilitate protein:protein interactions is thought to be site-specific. To better understand the ubiquitination status of the K-to-R single and double mutants, we analyzed Mcm10 that was overexpressed from a transgene (in wild-type cells) or expressed from its endogenous site. When Mcm10 was co-overexpressed with ubiquitin, ubiquitination was unaffected in the K85R mutant, but significantly diminished in the K372R and the KK85,372 RR double mutant (Fig. 3a). In contrast, when we analyzed endogenously expressed Mcm10 from cells that overexpressed ubiquitin, the mutations had very little effect on the ubiquitination status of the protein. Although the underlying reason for this discrepancy is not clear, we concluded that alternative ubiquitination sites must exist. To further corroborate this notion, we reanalyzed our mass spectrometry data using PEAKS® Studio software [24] and included protein fragments that were identified with a single peptide in one out of two experiments. This revealed four more ubiquitination sites at positions 112, 319, 414 and 436 (Fig. 3c).

### 3.4 The K372R mutation results in HU sensitivity when the checkpoint system is defective

**Fig. 4:**
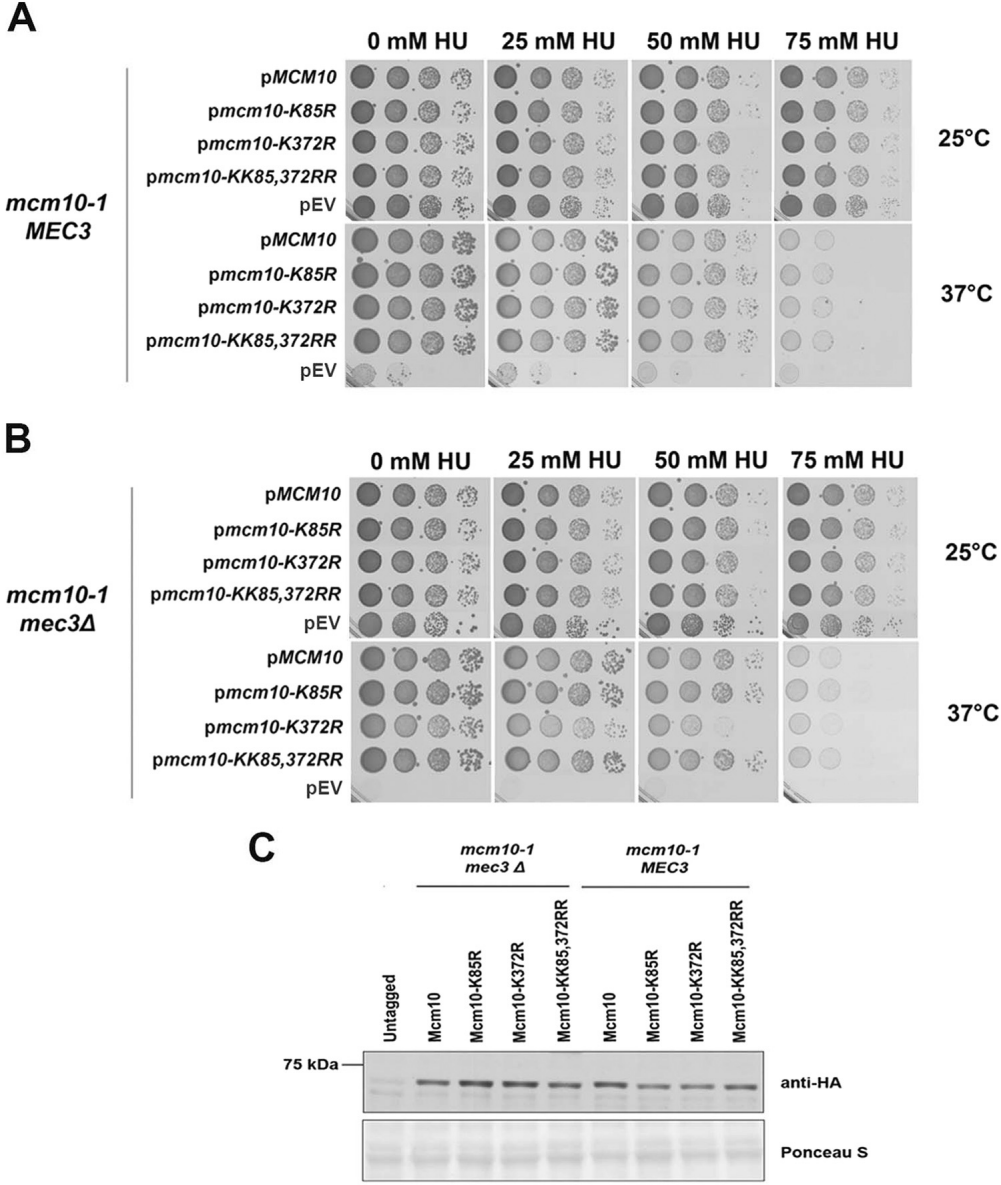
The Mcm10-K372R mutation sensitizes cells to replication stress when the 9-1-1 clamp is dysfunctional. (A) Serial, 10-fold dilutions of *mcm10-1* strains expressing plasmid-borne wild-type Mcm10 (p*MCM10*), or mutant forms (p*mcm10-K85R*, p*mcm10-K372R*, p*mcm10-KK85,372RR*), were grown for 3 days on SC-URA plates or SC-URA plates containing 25, 50 or 75 mM HU at 25 or 37 °C. Control strains expressed an empty vector (pEV). (B) Serial, 10-fold dilutions of *mcm10-1 mec3Δ* strains expressing plasmid-borne wild-type Mcm10 (p*MCM10*), or mutant forms (p*mcm10-K85R*, p*mcm10-K372R*, p*mcm10-KK85,372RR*), were grown for 3 days on SC-URA plates or SC-URA plates containing 25, 50 or 75 mM HU at 25 or 37 °C. Control strains expressed an empty vector (pEV). (C) Expression levels of plasmid-borne Mcm10 were analyzed by western blot. All constructs carried a 3HA tag. Whole cell extract was precipitated by TCA and protein levels were analyzed with an anti-HA antibody. Ponceau S staining served as a loading control.

To explore whether the K85R and K372R mutations caused any growth phenotype or sensitivity to the replication inhibitor hydroxyurea (HU), we complemented temperature-sensitive *mcm10-1* mutants with wild-type Mcm10, the K85R or K372R single mutant, or the KK85,372RR double mutant. All transgenes were expressed from a plasmid that contained the endogenous *MCM10* promoter [17]. Plasmids expressing wild-type or mutant *MCM10* were transformed into the *mcm10-1* strain and spotted on SC-URA plates in 10-fold dilutions. All four plasmids fully complemented the loss of Mcm10-1 protein at the nonpermissive temperature of 37 °C (Fig. 4a). Moreover, all four plasmids behaved similarly in the presence of HU (Fig. 4a). Nevertheless, it was possible that the K-to-R mutations caused problems during replication fork progression, resulting in ssDNA gaps. On the lagging strand, these gaps are sensed by the 9-1-1 checkpoint clamp. Thus, the presence of the checkpoint clamp may have masked the phenotype resulting from the K-to-R mutations. To test this hypothesis, we constructed strains that carried a deletion of *MEC3*, which encodes one of the subunits of the 9-1-1 checkpoint clamp. Since *MEC3* is not essential in *S. cerevisiae*, we knocked it out in the *mcm10-1* background. Plasmids expressing wild-type or mutant *MCM10* transgenes were transformed into *mcm10-1 mec3Δ* double mutants and spotted on SC-URA as described above. Surprisingly, in the presence of HU, the *mcm10-K372R* strain exhibited a significant growth defect at 37 °C when endogenous Mcm10-1 protein was degraded (Fig. 4b). *mcm10-K85R* and *mcm10-KK85,372 RR* mutants grew similarly to wild-type cells regardless of whether HU was present (Fig. 4b). Thus, the *mcm10-K372R* allele does not fully complement the *mcm10-1* growth defect under nonpermissive conditions (Fig. 4b). To ensure that plasmid borne Mcm10 was expressed at similar levels, we analyzed all strains by western blot.

## 4 Discussion

Ubiquitin regulates a wide variety of biological processes spanning protein degradation, cell signaling and protein:protein interaction. In yeast, the evolutionarily conserved replication factor Mcm10 is mono-ubiquitinated at two distinct lysines, and only the ubiquitinated form of the protein binds to PCNA [15]. Here, we mapped the ubiquitination sites of *S. cerevisiae* Mcm10. Evidence suggests that modifications of Mcm10 consistent with mono- and di-ubiquitination can also be detected in human cells [25].

Using MS analysis, we found that K85 and K372 are the two most prevalent sites of ubiquitination. The K372R mutation led to a notable decrease in Mcm10 ubiquitination when the protein was overexpressed in wild-type cells, indicating that this site is indeed a preferred attachment site for ubiquitin, whereas K85R is not. The fact that checkpoint deficient cells expressing Mcm10-K372R are sensitive to HU is indicative of a replication defect and may be due to dysregulation of the interaction between Mcm10 and PCNA. Although the ability to bind PCNA seems not significantly affected by the K372R mutation in a two-hybrid assay, it is conceivable that the interaction is suboptimal in the context of the replication fork.

It is intriguing that K372 is adjacent to the Zn-finger domain of Mcm10 [9]. Zn-fingers are known ubiquitination interacting motifs [26]. In Mcm10, the Zn-finger could bind intra-molecularly to ubiquitin at K372 and this could trigger a conformational shift, which could help to expose the PIP box of Mcm10 and make it accessible to PCNA. Either failure of anchoring PCNA at the right position or insufficient recruitment of PCNA may lead to elongation problems, which could be enhanced by the treatment with HU.

It was unexpected that *mcm10-K372*, but not *mcm10-K85R* and *mcm10-KK85,372RR* mutants showed enhanced sensitivity to HU in cells lacking the 9-1-1 checkpoint clamp. It appears that cells can use an alternative lysine (e.g., K122) together with K372 for ubiquitination when K85 is mutated. However, when K372 is mutated, it may be more difficult to find a “replacement” that can cooperate with ubiquitin at K85. When both K85 and K372 are mutated, cells find alternative sites for ubiquitination that work as well as the K85-K372 combination, and restore the biological function of di-ubiquitinated Mcm10.

Although we cannot formally exclude that the K372R phenotype is caused by other defects unrelated to ubiquitination, we deem this possibility highly unlikely. K372 is evolutionarily not conserved and has not been implicated in other protein interactions or DNA binding [9]. Lastly, the interaction between Mcm10 and PCNA is essential for DNA replication, and the observed redundancy for ubiquitination can be interpreted as a mutation buffer. Importantly, ubiquitin attachment is not completely random. Out of 63 lysines in Mcm10, we only found a subset of six to be target sites for ubiquitination, arguing that site specificity is important.

## Conflict of interest

The authors declare that they don’t have any conflict of interest.

## Footnotes

Appendix A Supplementary data associated with this article can be found in the online version at doi:10.1016/j.bbrep.2016.09.003.
