# Supplementary material for: Mapping ubiquitination sites of S. cerevisiae Mcm10
Source: Biochem Biophys Rep. 2016 Sep 19;8:212–8. doi: 10.1016/j.bbrep.2016.09.003 (PMC5421568; doi:10.1016/j.bbrep.2016.09.003)
Supplement: Supplementary file 5 — Supplementary material [file mmc5.pdf]

SUPPLEMENTARY MATERIAL

Table S1, related to 2. Materials & methods, yeast strains generated in this study

**Strain name**	**Relevant genotype**	**Source**
Strains derived from (W303-1a)	*MATa ura3-1 ade 2-1 his3-11,-15 leu2-3,-112 can1-100 trp1-1 BAR1::LEU2 rad5-535*	Das-Bradoo *et al*., 2006[1]
ABy1007	p*YES2-NT/B, YEp96-CUP1-non-tagged-UBIQUITIN*	This study
ABy1334	p*YES2-GAL-6HIS-MCM10-3HA YEp105-CUP1-UBIQUITIN*	This study
ABy1689	p*YES2-GAL-6HIS-mcm10-K85R-3HA YEp105-CUP1-UBIQUITIN*	This study
ABy1690	p*YES2-GAL-6HIS-mcm10-K372R-3HA YEp105-CUP1-UBIQUITIN*	This study
AByb1691	p*YES2-GAL-6HIS-mcm10-KK85,372RR-3HA YEp105-CUP1-UBIQUITIN*	This study
AByb1925	*mcm10-1, MEC3::TRP,* pRS316-*MCM10-2HA*	This study
AByb1926	*mcm10-1, MEC3::TRP,* pRS316-*mcm10-K85R-2HA*	This study
AByb1927	*mcm10-1, MEC3::TRP,* pRS316-*mcm10-K372R-2HA*	This study
AByb1928	*mcm10-1, MEC3::TRP,* pRS316-*mcm10-KK85,372RR-2HA*	This study

AByb1942	*mcm10-1,* pRS316-*MCM10-2HA*	This study
AByb1943	*mcm10-1,* pRS316-*mcm10-K85R-2HA*	This study
AByb1944	*mcm10-1,* pRS316-*mcm10-K372R-2HA*	This study
AByb1945	*mcm10-1,* pRS316-*mcm10-KK85,372RR-2HA*	This study
AByb2339	*MCM10::MCM10-3HA-8HIS*	This study
AByb2341	*MCM10::mcm10-K85R-3HA-8HIS*	This study
AByb2343	*MCM10::mcm10-K372R-3HA-8HIS*	This study
AByb2345	*MCM10::mcm10-KK85,372RR-3HA-8HIS*	This study
AByb2352	*MCM10::MCM10-3HA-8HIS, MEC3::HIS3*	This study
AByb2353	*MCM10::mcm10-K85R-3HA-8HIS, MEC3::HIS3*	This study
AByb2354	*MCM10::mcm10-K372R-3HA-8HIS, MEC3::HIS3*	This study
AByb2355	*MCM10::mcm10-KK85,372RR-3HA-8HIS, MEC3::HIS3*	This study
ABy2389	*MCM10, YEp105-CUP1-UBIQUITIN*	This study
AByb2391	*MCM10::MCM10-3HA-8HIS, YEp105-CUP1-UBIQUITIN*	This study
AByb2393	*MCM10::mcm10-K85R-3HA-8HIS, YEp105-CUP1-UBIQUITIN*	This study
AByb2394	*MCM10::mcm10-K372R-3HA-8HIS, YEp105-CUP1-UBIQUITIN*	This study
AByb2395	*MCM10::mcm10-KK85,372RR-3HA-8HIS, YEp105-CUP1-UBIQUITIN*	This study

[1] Das-Bradoo, S., R.M. Ricke, and A.K. Bielinsky, **Interaction between PCNA and diubiquitinated Mcm10 is essential for cell growth in budding yeast.** Mol Cell Biol, 26 (2006), pp. 4806-4817
